# Supplementary material for: Medication-related hospital readmissions within 30 days of discharge—A retrospective study of risk factors in older adults
Source: PLoS One. 2021 Jun 10;16(6):e0253024. doi: 10.1371/journal.pone.0253024 (PMC8191889; doi:10.1371/journal.pone.0253024)
Supplement: S1 Appendix — Includes the AT-HARM10 assessment tool, instructions for use and representative examples of when a question should be answered "Yes" or "No". (DOCX) [file pone.0253024.s001.docx]

**AT-HARM10 – Instructions**

**Assessment Tool for identifying Hospital Admissions Related to Medications**

The Assessment Tool for identifying Hospital Admissions Related to Medications (AT-HARM10) is a screening tool consisting of ten questions used to determine whether a hospital admission is medication-related. A medication-related admission (MRA) is a hospital admission of which a medication related problem (MRP) is either the main cause for admission or a significantly contributing cause for admission (i.e. without the MRP, the patient would not have been admitted). MRPs are defined here as “undesirable patient experiences that involve medication therapy and that actually or potentially interfere with desired patient outcomes”. These not only involve adverse drug reactions to prescribed medication, but can also involve problems such as inappropriate prescribing and non-compliance, and problems related to over-the-counter (OTC) medications. It does not consider whether the admission was preventable (e.g. an admission caused by side effects of appropriate medication treatment is considered medication-related). AT-HARM10 was developed to measure the incidence of possibly medication-related admissions, MRAs.

The user of AT-HARM10 should not have to go through all patient data in the patient’s medical record, because that would take too much time. The patient data from the medical records that will be provided for the assessment include: admission notes from the current admission, medication list, laboratory data, pharmacists’ notes and the discharge summary for the admission. All registered medications, including over-the-counter (OTC) medication, should be considered in the assessment. Non-registered complementary and alternative medicine (CAM) products and dietary supplements are not to be considered.

The tool comprises ten questions which can only be answered "Yes" or "No". For further clarification of each question, please see the examples below. Questions 1-3 are used to identify admissions that are unlikely to be medication-related (U), while questions 4-10 are used to identify possibly medication-related (P) admissions. The assessment is finished as soon as the answer "Yes" is given for any question, resulting in the admission being either U or P. This means that it is not necessary to answer the remaining questions when a “Yes” answer has been given. If all the questions are answered "No", the assessment is still indecisive and needs to be examined by an expert panel.

Please note: While the reason for visiting the emergency department (ED) might be non-medication-related (e.g. chest pain, head ache), in some cases the primary cause for admission might turn out to be medication-related (e.g. low potassium levels discovered while at the ED – worsened by a diuretic). In these cases, the admission should be classified as P.

**AT-HARM10**

**Assessment Tool for identifying Hospital Admissions Related to Medications**

**Note**: Questions 1-3 are used to identify admissions unlikely to be medication-related, while questions 4-10 are used to identify possibly medication-related admissions. The assessment is finished as soon as the answer "Yes" is given for any question 🡪 U (unlikely to be medication-related) or P (possibly medication-related). If all the questions are answered with "No", the admission should be classified as P (possibly medication-related).

1. Was the admission caused by an *infection* or a previously *undiagnosed* disease (e.g. diabetes or heart failure) that is *not medication-related*?

Yes 🡪 U (unlikely to be medication-related)

No 🡪 NQ (next question)

1. Was the admission caused by progression of a previously diagnosed disease that is *not medication-related* (with the progression of several chronic diseases, such as congestive heart failure or diabetes, a medication-related component can rarely be excluded)?

Yes 🡪 U

No 🡪 NQ

**NOTE**: Appropriateness of medication treatment should only be considered in relation to this question to determine whether the admission is primarily caused by disease progression (*unlikely* MRA) or suboptimal medication treatment or use (*possible* MRA, question 4-10).

1. Was the admission caused by physical trauma, substance intoxication, social circumstances or allergies (e.g. car accident, wasp allergy, alcohol excess, mushroom poisoning) that are *not medication-related*?

Yes 🡪 U

No 🡪 NQ

1. Is it hinted or stated in the medical record that the admission was *medication-related* (including non-compliance)?

Yes 🡪 P (possibly medication-related)

No 🡪 NQ

1. Might (side) effects of the medications the patient was taking (prescribed or non-prescribed) prior to hospitalisation have caused the admission (including over-treatment)?

Yes 🡪 P

No 🡪NQ

**NOTE**: An admission caused by side effects of appropriate medication treatment should be classified as *possibly* medication-related.

1. Are there abnormal laboratory results or vital signs that could be *medication-related* and might have caused the admission?

Yes 🡪 P

No 🡪 NQ

1. Was there any drug-drug interaction or drug-disease interaction (i.e. a contraindication) that might have caused the admission?

Yes 🡪 P

No 🡪 NQ

1. Did the patient have any *previously* diagnosed untreated or suboptimally treated (e.g. dose too low) indications that might have caused the admission?

Yes 🡪 P

No 🡪 NQ

1. Was the patient admitted because of a problem with the dosage form or pharmaceutical formulation (i.e. failure to receive the medication)?

Yes 🡪 P

No 🡪 NQ

1. Is the cause of the admission a response to cessation or withdrawal of medication therapy?

Yes 🡪 P

No 🡪 P (the tool has not been able to rule out that the admission is medication-related)

**AT-HARM10 – Examples**

**Assessment Tool for identifying Hospital Admissions Related to Medications**

Representative examples of when a question should be answered "Yes" or "No".

1. Was the admission caused by an *infection* or a previously *undiagnosed* disease (e.g. diabetes or heart failure) that is *not medication-related*?

**Yes**: A patient admitted because of pneumonia that was *not related* to the patient's *medications*.

**Yes**: A patient admitted because of rectal bleeding found, after investigation, to have been caused by a tumour.

**Yes**: A patient admitted with unclear diagnosis and new symptoms. The symptoms cannot be explained by the patient’s current medications.

**No**: A patient receiving immunosuppressive treatment admitted with infection.

**No**: A patient admitted with new symptoms indicating heart failure (oedema, shortness of breath) and a history of excessive use of non-steroidal anti-inflammatory drugs (NSAIDs).

1. Was the admission caused by progression of a previously diagnosed disease that is *not medication-related*?

**NOTE**: Appropriateness of medication treatment should only be considered in relation to this question to determine whether the admission is primarily caused by disease progression (*unlikely* MRA) or suboptimal medication treatment or use (*possible* MRA, question 4-10).

**Yes**: A patient admitted because of progression of cancer that is not related to the patient's medications.

**Yes:** A patient admitted because of exacerbation of congestive heart-failure, which worsened despite optimal treatment (the medication therapy seems to follow the applicable treatment guidelines) and with no signs of non-compliance.

**No**: A diabetic patient admitted because of hyperglycaemia without other reason for admission (hyperglycaemia should never lead to admission in a patient that is optimally treated).

1. Was the admission caused by physical trauma, substance intoxication, social circumstances or allergies (e.g. car accident, wasp allergy, alcohol excess, mushroom poisoning) that are *not medication-related*?

**Yes**: A patient admitted because of alcohol intoxication or a car accident that was *not related* to the use of the patient's *medications*.

**No**: A patient admitted because of alcohol intoxication worsened by the concomitant use of sedatives.

1. Is it hinted or stated in the medical record that the admission is *medication-related* (including non-compliance)?

**Yes**: A physician states in the discharge note that the patient was admitted because of constipation caused by the lack of laxative therapy during treatment with a strong opioid.

**Yes**: A patient admitted because of an epileptic seizure and a note in the medical records that the patient is known to be non-compliant.

1. Might (side) effects of the medications the patient was taking (prescribed or non-prescribed) prior to hospitalisation have caused the admission (including over-treatment)?

**NOTE**: An admission caused by side effects of appropriate medication treatment should be classified as *possibly* medication-related.

**Yes**: A patient admitted with a gastric bleeding who uses acetyl salicylic acid to prevent thrombotic events (regardless of the presence of a correct indication and of the use of a proton pump inhibitor for gastric protection).

**Yes**: A patient admitted because of lactic acidosis after continuing medication with metformin while experiencing dehydrating stomach flu.

**Yes**: A patient who uses antihypertensive medication and was admitted due to a fall caused by orthostatic hypotension.

1. Are there abnormal laboratory results or vital signs that could be *medication-related* and might have caused the admission?

**Yes**: A patient admitted with a serum digoxin concentration of 3.4 nmol/L (toxic concentration) which may have been the cause for admission.

**Yes**: A patient admitted because of hypokalaemia (s-potassium < 3.5 mmol/L) and prescribed a diuretic.

**Yes**: A patient with epilepsy admitted with seizures and prescribed a seemingly adequate dose of carbamazepine but with a measured plasma concentration that is too low.

1. Was there any *drug-drug interaction* or *drug-disease interaction* (i.e. a contraindication) that might have caused the admission?

**Yes**: A patient admitted because of gastrointestinal bleeding who was taking diclofenac and warfarin in combination before admission.

**Yes**: A patient admitted because of serotonin syndrome who was taking tramadol, citalopram and mirtazapine.

**Yes**: A patient, previously diagnosed with bilateral renal artery stenosis, admitted because of acute renal failure after taking an ACE inhibitor.

**Yes**: A patient with dementia, who has recently been prescribed an anticholinergic medication (e.g. hydroxyzine), admitted with confusion.

1. Did the patient have any, *previously* diagnosed, untreated or suboptimally treated (e.g. dose too low) indications that might have caused the admission?

**Yes**: A patient diagnosed with congestive heart failure, who was taking only a starting dose of ACE-inhibitor (unjustifiably low dose), admitted because of fluid retention and dyspnoea.

**Yes**: A patient admitted because of a hip fracture who had a prior diagnosis of osteoporosis but was not taking osteoporosis prophylaxis.

1. Was the patient admitted because of a problem with the dosage form or pharmaceutical formulation (i.e. failure to receive the medication)?

**Yes**: A patient admitted with worsening asthma who was found to be unable to use the inhalers correctly.

**Yes**: A patient admitted with palpitations who was found to be unable to swallow tablets and had been crushing slow-release antihypertensive tablets that should have been swallowed whole to retain their slow-release effects.

1. Is the cause of the admission a response to cessation or withdrawal of medication therapy?

**Yes**: A patient whose prednisolone treatment has been discontinued too abruptly admitted with nausea, vomiting and diarrhoea.
