# Supplementary material for: Medication-related hospital readmissions within 30 days of discharge—A retrospective study of risk factors in older adults
Source: PLoS One. 2021 Jun 10;16(6):e0253024. doi: 10.1371/journal.pone.0253024 (PMC8191889; doi:10.1371/journal.pone.0253024)
Supplement: Attachment — Submitted filename: Response to reviewers Glans Maria.docx [file pone.0253024.s002.docx]

May 18, 2021

**Manuscript ID: PONE-d-21-08047**

**Medication-related hospital readmissions within 30 days of discharge – a retrospective study of risk factors in older adults**

**PLOS ONE**

Dear reviewer,

Thank you for your feedback and suggestions of ways to strengthen our paper further. We have attempted to address the comments below with the hope that the manuscript has improved enough to make acceptance possible.

Yours sincerely,

Maria Glans

(for all the co-authors)

Lund University

Center for Primary Health Care Research

Box 50332, SE-202 13 Malmö, Sweden

E-mail: [maria.glans@med.lu.se](mailto:maria.glans@med.lu.se)

**Reviewers’ comments**

**Overall, the authors provide data that healthcare systems and health policy makers could potentially use in improving care for older patients who are hospitalized and discharged back into the community. While I recommend accepting this manuscript for publication, improvements could be made by addressing the following points.**

1. **It is unclear why the authors did not match the two cohorts by the admitting hospital department (e.g., General Surgery, Gynaecology, etc). It is possible that patients admitted by differing departments have systematically different distribution of risk factors. Inclusion of the distribution of admitting department between the two cohorts in Table 1 would be informative. If differences are stark, then adjustments in the logistic models would be suggested.**

*Thank you for this insightful comment. Unfortunately, since the study is based on data retrieved from a larger study, we were not able to match the two cohorts. However, since the study is focusing on risk factors of readmission connected to the initial hospital visit, we have compared the groups (readmitted patients vs comparison group and possibly medication-related readmissions vs unlikely medication-related readmissions) regarding discharging unit at initial hospital discharge and found no significant differences between groups. This is now clarified in lines 208-209 as well as in Table 2 in the manuscript.*

2. **The authors state (Lines 144-145) that data were collected in a standardized way. It would be informative to know how the authors prevented differential assessment of risk factors based on the study outcome (i.e., whether/how the abstractor was blinded to the readmission status)?**

*Thank you. It would indeed have been good to have blinded the review of medical records as well as the assessment of risk factors, but this was not done in this study. Objectivity was, naturally, sought at all points. We have now tried to clarify this in line 145 and 149. We have also added it as a limitation in the section Strengths and limitations (line 364-366).*

3. **The authors state (Lines 158-159) that if all questions were answered ‘No’ that the admission was classified as possibly medication-related. Should this be ‘unlikely medication related’ instead?**

*Thank you. To answer this question we kindly refer to the S1 Appendix – AT-HARM10 where it is stated “If all the questions are answered with “No”, the admission should be classified as P (possibly medication related)” – see clipping below:*

*However, the instructions to the AT-HARM10 instrument further states:* If all the questions are answered "No", the assessment is still indecisive and needs to be examined by an expert panel.

*Luckily, in none of the included readmissions all the questions were answered “No”, hence no further examination was needed. We have tried to further clarify this in lines 200-201.*
